# Enhancement of acousto-optic interaction using a phoxonic cavity with structural hierarchy

**DOI:** 10.1038/s41598-024-57816-2

**Published:** 2024-04-16

**Authors:** Junyong An, Seongmin Park, Wonju Jeon

**Affiliations:** Department of Mechanical Engineering, Korea Advanced Institute of Science and Technology, 291 Daehak-ro, Yuseong-gu, Daejeon, 34141 Republic of Korea

**Keywords:** Sound, Infrared light, Acousto-optic interaction, Phoxonic cavity, Phononic and photonic crystals, Acoustics, Photonic crystals

## Abstract

We propose a phoxonic cavity with structural hierarchy to enhance acousto-optic interaction in acoustically dissipative media. In a conventional phoxonic cavity, interaction between infrared light and hypersound with the same wavelength scale became weak due to large acoustic attenuation whose coefficient is proportional to the square of the frequency. To alleviate the acoustic attenuation, it is necessary to use low-frequency sound with much longer wavelength than the infrared light, but the conventional phoxonic cavity is not suitable for confining such hypersound and infrared light simultaneously. In this study, we employ the concept of structural hierarchy into the phoxonic cavity to control infrared light and hypersound with different wavelength scales. A phoxonic cavity with two different scales achieves the acousto-optic interaction approximately 1.6 times that in the conventional one. To further enhance the interaction, we adjust geometrical constitution and material properties of the two-scale phoxonic cavity using quasi-static homogenization theory, leading to the interaction about 2.1 times that in the conventional cavity.

## Introduction

The fields of optics and acoustics each have long histories, and each field has experienced tremendous development. However, the study of phenomena that involve an interaction between light and sound, where one wave affects the behavior of the other, has a relatively short history. In 1922, Léon Brillouin^1^ theoretically predicted that a change in the refractive index due to sound passing through a medium diffracted a light beam. Ten years later, the predicted phenomenon was experimentally demonstrated by Debye and Sears^2^. Since then, the interaction phenomenon has received increasing attention because of its applications such as modulator^3^, filter^4,5^, field reconstruction^6,7^, beamformer^8^, and Bragg imaging^9,10^, optical pulse storage^11^, optical information conversion^12^, and phonon lasers^13^.

Several researchers have focused on enhancing acousto-optic (AO) interactions^14–18^ because the enhancement is conducive to improving performance of the abovementioned devices and techniques. Slowing the speed of light is one efficient way to enhance AO interactions. For example, ultraslow light in a dielectric fiber doped with three-level Λ-type ions yielded phase-matching conditions, allowing researchers to achieve strong interaction between light and sound confined in the fiber^14^. In addition, the slow-moving light increases the interaction time, enhancing the AO interaction. Courjal et al.^15^ used photonic crystals to increase the duration of the AO interaction at frequencies near the bandgap. As a result, the acousto-optic interaction drove a change in the effective refractive index that was up to 61 times larger than that without photonic crystals.

It was recently reported that enhancement of AO interaction was achieved using a phoxonic cavity that confines sound and light in the same region^16–18^. The phoxonic cavity induced Fabry-Pérot resonance confining the two waves with wavelengths of the same order of magnitude. For example, sound at GHz frequencies and 1550 nm light, commonly used in telecommunications, were confined and interacted with each other in the phoxonic cavity^19^. However, this high-frequency sound was attenuated significantly in acoustically dissipative media because its acoustic attenuation coefficient is proportional to the square of the acoustic frequency^18^.

Acoustic attenuation due to media loss is negligible when using sound with frequencies in hundreds of MHz, but the conventional phoxonic cavity cannot confine both infrared light and hypersound with different wavelength scales. To control the infrared light and hypersound, in this study, we propose a phoxonic cavity with structural hierarchy. Geometrical concept of the structural hierarchy has been employed to improve functionalities of metamaterials and photonic crystals^20–23^.﻿ The proposed phoxonic cavity is composed of phoxonic crystals on the scale of the acoustic wavelength, photonic crystals on the scale of the optical wavelength, and a cavity. A challenge on confining the two waves with different wavelengths is tackled by utilizing the proposed phoxonic cavity, resulting in enhancement of AO interaction.

The remainder of this paper is organized as follows. In section “Phoxonic cavity with structural hierarchy”, we describe geometrical constitution and material properties of the proposed phoxonic cavity with structural hierarchy, and examine its ability to confine the two waves with different wavelengths by calculating the displacement and electric fields of the phoxonic cavity theoretically. In section “Acousto-optic interactions in phoxonic cavities with structural hierarchy”, we demonstrate that the proposed phoxonic cavity achieves stronger AO interaction than the conventional phoxonic cavity. To further enhance the interaction, we modify the geometrical constitution and material properties of the phoxonic cavity via quasi-static homogenization theory. Section “Conclusions” provides the conclusions of the study.

## Phoxonic cavity with structural hierarchy

In this section, we propose a phoxonic cavity with structural hierarchy to control acoustic and optical waves with different wavelength scales. First, we explain the terms “phoxonic crystal” and “phoxonic cavity”. We then describe the geometry and material of the proposed phoxonic cavity. Next, we calculate the displacement and electric fields of the proposed phoxonic cavity using the transfer matrix method^24,25^. By comparing these fields, we demonstrate that structural hierarchy is conducive to confining hypersound and infrared light in a cavity.

The phoxonic crystal is a periodic structure that manipulate sound and light simultaneously by exhibiting acoustic and optical bandgaps^26,27^. That is, the phoxonic crystal performs the functions of phononic and photonic crystals; the ‘x’ in the ‘phoxonic’ represents the ‘n’ in ‘phononic’ and the ‘t’ in ‘photonic’ simultaneously. The phoxonic cavity is a phoxonic crystal that contains a cavity. Incident optical and acoustic waves are confined in the cavity at certain frequencies within bandgaps due to Fabry–Perot resonance.

**Figure 1:**
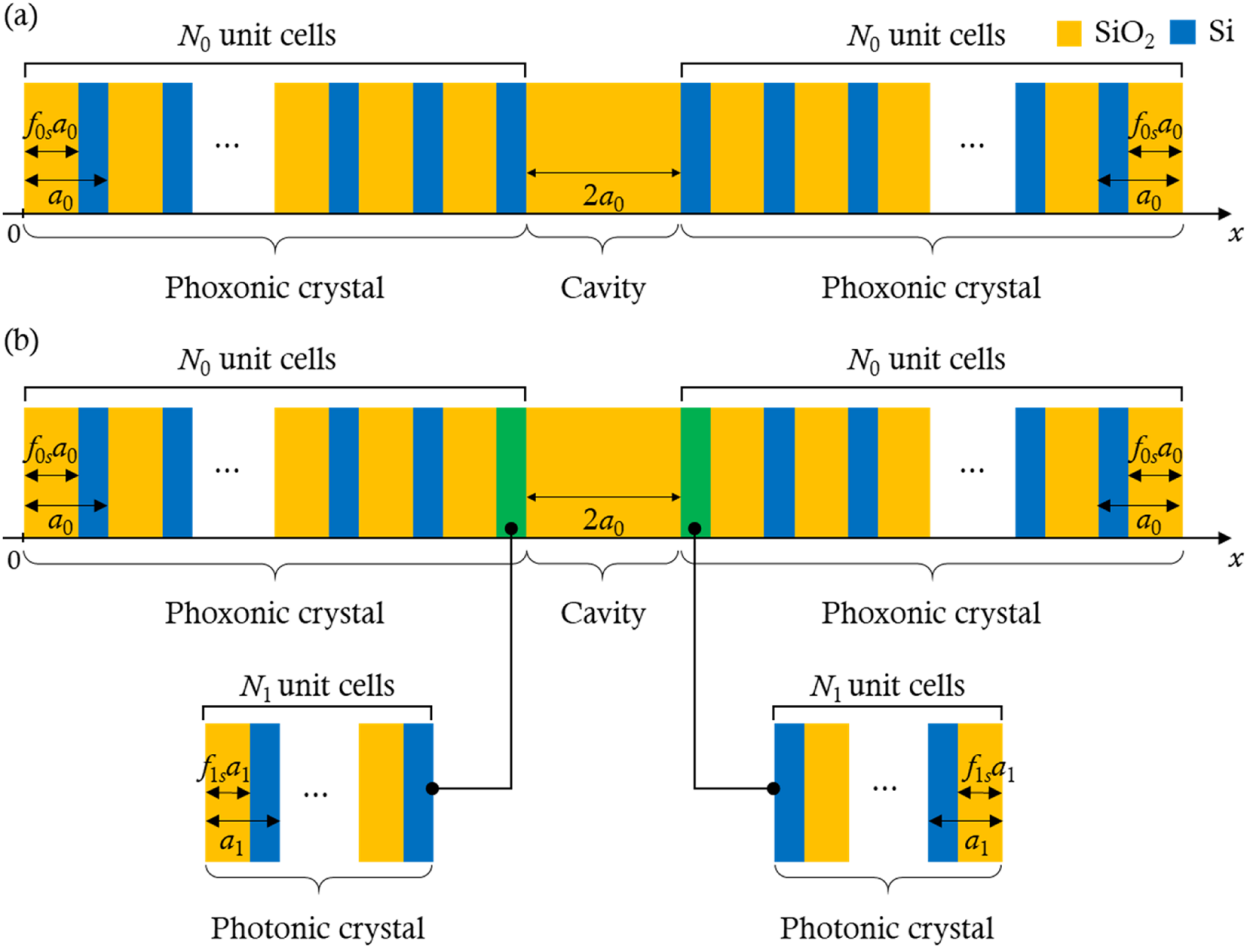
Geometries and materials of the (**a**) single-scale phoxonic cavity^16,18^ and (**b**) two-scale phoxonic cavity. In (**a**), ${N}_{0}$ is the number of unit cells in the phoxonic crystal, ${a}_{0}$ is the size of the unit cell, and ${f}_{0s}$ is the SiO_2_ filling fraction within the unit cell. The size of the SiO_2_ cavity is $2{a}_{0}$. In (**b**), the photonic crystals are composed of SiO_2_ and Si layers with filling fractions of ${f}_{1s}$ and ${1-f}_{1s}$, respectively. The number and the size of the unit cell in a photonic crystal are ${N}_{1}$ and ${a}_{1}$, respectively. The material properties of Si and SiO_2_ are listed in Table 1.

Figure 1a shows the geometry of one-dimensional (1-D) phoxonic cavity that has been studied previously^16,18^. The phoxonic cavity contains two phoxonic crystals composed of silicon (Si)—silica (SiO_2_) layers that are mirror-symmetric with respect to the SiO_2_ cavity. We present the geometry of the 1-D phoxonic cavity with structural hierarchy in Fig. 1b by replacing the two Si layers (blue layers) adjacent to the cavity with photonic crystals (green layers) composed of ${N}_{1}$ unit cells. While the phoxonic cavity in Fig. 1a contains unit cells with a single scale, there are two different unit cell scales in the phoxonic cavity with structural hierarchy. In this paper, the phoxonic cavities in Fig. 1a and b are referred to as single-scale and two-scale phoxonic cavities, respectively.

**Table 1:** The material properties of Si and SiO_2_.

Material	$n$	$p$	$\rho ({\text{kg}}/{{\text{m}}}^{3})$	${c}_{s} ({\text{m}}/{\text{s}})$	$\alpha /{f}^{2} ({{\text{s}}}^{2}/{\text{m}})$
Si	3.46	0.01	2330	8430	1.07 × 10^–16^
SiO_2_	1.46	0.27	2200	5970	5.86 × 10^–16^

Here, $n$ is the refractive index, $p$ is the photoelastic coefficient, $\rho$ is the mass density, ${c}_{s}$ is the speed of sound, $\alpha$ is the acoustic attenuation coefficient^28,29^, and $f$ is the acoustic frequency.

**Figure 2:**
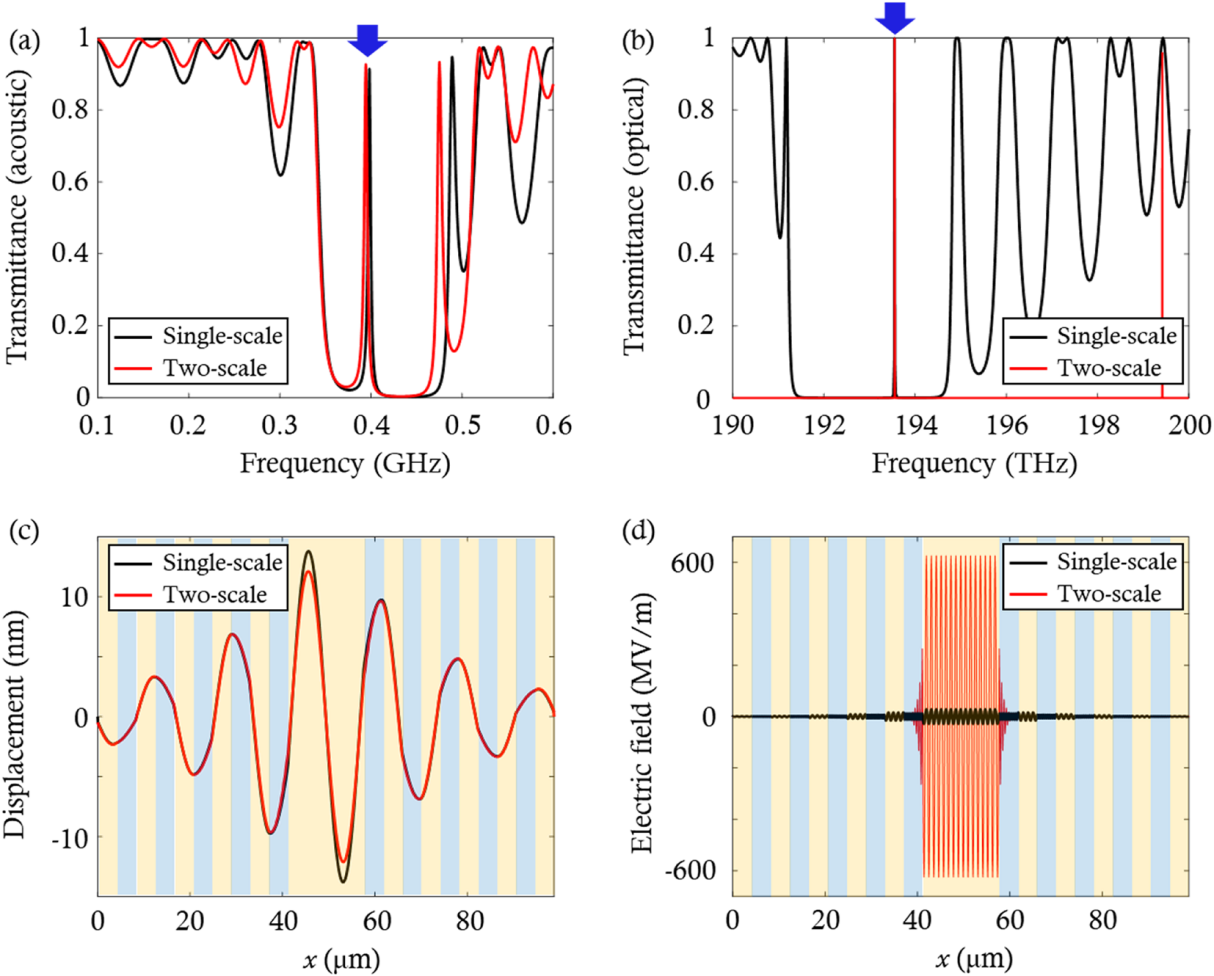
(**a**) Acoustic and (**b**) optical transmittance spectra of the phoxonic cavities in Fig. 1. The blue arrows in (**a**) and (**b**) indicate the acoustic and optical cavity modes, respectively. (**c**) Displacement fields of the phoxonic cavities at the acoustic cavity mode frequency. (**d**) Electric fields of the phoxonic cavities at the optical cavity mode frequency. In (**c**) and (**d**), the orange and blue regions represent the SiO_2_ and Si layers, respectively.

The phoxonic cavities, which exhibit both phononic and photonic bandgaps, induce the localized cavity modes within these bandgaps. It is necessary to produce the localized cavity modes at target frequencies to efficiently modulate infrared light by hypersound. Here, we adjust the geometrical parameters of the phoxonic cavities so that the cavity modes appear at the acoustic and optical target frequencies of 0.4 GHz and 193.5 THz, respectively. The values of ${a}_{0}$ and ${f}_{1s}$ are swept in the range of 100 nm $\le {a}_{0}\le$10,000 nm and 0 $\le {f}_{1s}\le$ 1, with increments of 10 nm and 0.001, respectively. The other parameters are fixed at ${f}_{0s}=0.5$, ${N}_{0}=5,$ ${N}_{1}=8$, and ${a}_{1}=0.514$. When the geometrical parameters (${a}_{0}, {a}_{1}, {f}_{0s}, {f}_{1s}, {N}_{0}, {N}_{1}$) are set to (8.23 μm, 0.514 μm, 0.5, 0.514, 5, 8), the acoustic and optical cavity modes occur at the target frequencies. To demonstrate these cavity modes, the acoustic and optical transmittance spectra of the phoxonic cavities are calculated using the transfer matrix method^24,25^ and are shown in Fig. 2a and b. The solid black and red lines indicate the spectra of the single- and two-scale phoxonic cavities, respectively. In Fig. 2a and b, peaks due to the Fabry–Perot resonance appears at 0.4 GHz and 193.5 THz within the bandgaps, as indicated by the blue arrows.

We now examine the confinement of waves in the phoxonic cavities by calculating their displacement and electric fields. Figure 2c illustrates the displacement fields of the single- (solid black line) and two-scale (solid red line) phoxonic cavities at the acoustic cavity mode frequency when the left end of the phoxonic cavity is excited using an input strain level of 0.1%. In the displacement fields, the amplitude of the displacement field is maximized in the SiO_2_ cavity because the incident acoustic wave is localized in it via Fabry–Perot resonance. The maximum amplitude becomes slightly smaller in a two-scale phoxonic cavity than in a single-scale phoxonic cavity. This is because the photonic crystals adjacent to the cavity have material properties different from those of Si, so the periodicity of the phoxonic crystal unit-cell is broken. Nevertheless, the two-scale phoxonic cavity still has a good capability to confine the acoustic wave in the cavity, as shown in Fig. 2c.

Figure 2d shows the electric fields at the optical cavity mode frequency for the single-scale (solid black line) and two-scale (solid red line) phoxonic cavities when the magnitude of the incident electric field is 1 MV/m. The maximum value of electric field within the two-scale phoxonic cavity is 20 times as large as that of the single-scale phoxonic cavity when $x$ ranges from approximately 40–60 μm. This is because the photonic crystals act as optical mirrors with high reflection coefficients for 1550 nm light, resulting in high confinement of optical waves in the cavity.

With the aid of this structural hierarchy, the ability to confine optical waves was increased significantly while the ability to confine acoustic waves was reduced slightly. The proposed phoxonic cavity with structural hierarchy is suitable for confining hypersound and infrared light with different wavelength scales in the cavity. In the following section, we quantitatively compare the acousto-optic interaction between single and two-scale phoxonic cavities.

## Acousto-optic interactions in phoxonic cavities with structural hierarchy

### Enhancement of acousto-optic interaction using the two-scale phoxonic cavity

In this section, we examine AO interactions in the single- and two-scale phoxonic cavities. In order to quantify the degree of AO interaction, we evaluate modulation of the optical cavity mode frequency^17,30, 31^. By comparing the modulations between single- and two-scale phoxonic cavities, we demonstrate that the proposed phoxonic cavity is useful for enhancing AO interaction.

Two effects contribute to modulation of the optical cavity mode frequency. One is the photo-elastic (PE) effect and the other is the moving interface (MI) effect^30,31^. In the former, mechanical deformation in the phoxonic cavity due to acoustic waves changes the dielectric permittivities of the silicon (Si) and silica (SiO_2_) layers, modulating the cavity mode frequency. In the latter, the moving interfaces (MI) between the Si and SiO_2_ layers cause variations in the dielectric permittivity near the interfaces, modulating the cavity mode frequency.

For the single- and two-scale phoxonic cavities, frequency modulations of optical cavity mode due to the PE (${g}_{PE}$) and MI (${g}_{MI}$) effects are expressed as follows:^30,31^

1 $${g}_{PE}=-\frac{\omega }{2}\frac{{\int }_{0}^{\left(2{N}_{0}+2\right){a}_{0}}p\left(x\right)\varepsilon \left(x\right){n}^{2}\left(x\right)\frac{\partial u\left(x\right)}{\partial x}{\left(E\left(x\right)\right)}^{2}dx}{{\int }_{0}^{\left(2{N}_{0}+2\right){a}_{0}}\varepsilon \left(x\right){\left(E\left(x\right)\right)}^{2}dx},$$

2 $${g}_{MI}=-\frac{\omega }{2}\frac{\sum_{j=1}^{{N}_{t}}(\varepsilon ({x}_{j}^{+})-\varepsilon ({x}_{j}^{-}))u({x}_{j}^{-}){\left(E\left({x}_{j}^{-}\right)\right)}^{2}}{\int \varepsilon \left(x\right){\left(E\left(x\right)\right)}^{2}dx}.$$

Here, $\omega$ is the angular frequency of the optical cavity mode; $p(x)$, $\varepsilon (x)$ and $n\left(x\right)$ denote respectively the photoelastic coefficient, the electric permittivity and the refractive index of the phoxonic cavities at point $x$; $u(x)$ and $E\left(x\right)$ are the displacement and electric fields within the phoxonic cavities, respectively; ${x}_{j}$ is the position of the right-side boundary of the $j$th layer from the left end of each phoxonic cavity; ${x}_{j}^{+}$ and ${x}_{j}^{-}$ are the positions of the right- and left-side boundaries, respectively, of the $j$th layer; and ${N}_{t}$ is the total number of layers. We also calculate the total modulation (${g}_{t}$), which results from both the photoelastic effect and moving interface effect, and the modulation (${g}_{t}$) is obtained by

3 $${g}_{t}={g}_{PE}+{g}_{MI}$$

For single- and two-scale phoxonic cavities, the values of ${g}_{PE}$ and ${g}_{MI}$ are obtained by using Eqs. (1) and (2) with the displacement ($u(x)$) and electric ($E(x)$) fields shown in Fig. 2c and d. By incorporating the concept of structural hierarchy into the single-scale phoxonic cavity, we could obtain increased values of (${g}_{PE}$, ${g}_{MI}$, ${g}_{t}$) from (43 GHz, 428 GHz, 471 GHz) to (143 GHz, 625 GHz, 768 GHz). It is noted that the total frequency modulation (${g}_{t}$) is increased by 63%, which is approximately 1.6 times that in the single-scale phoxonic cavity. These results indicate that the acoustic and optical waves with different wavelength scales interact with each other more strongly in the proposed two-scale phoxonic cavity than in the single-scale phoxonic cavity.

### Further enhancement of AO interaction by modifying the geometrical constitution

While the AO interaction was enhanced in the previous section by using the two-scale phoxonic cavity, the ability to confine the acoustic wave was reduced slightly. This is because the photonic crystals have effective material properties (mass density and Young’s modulus) that are different from those of silicon (Si). This breaks the periodicity of the acoustic mirrors. If the effective material properties of the photonic crystals are adjusted more similar to those of Si, the ability to confine the acoustic wave can be increased further. In this section, we modify the geometrical constitution of the photonic crystals to make their effective material properties more similar to those of Si, in order to enhance AO interaction further.

**Figure 3:**
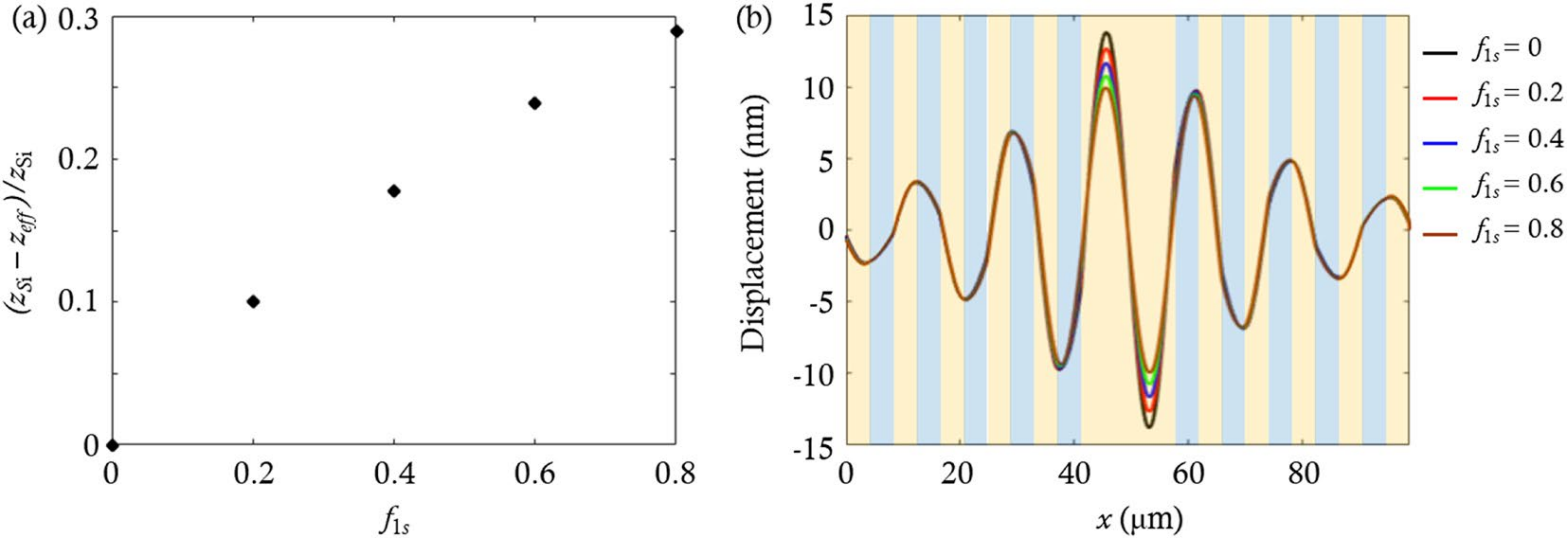
(**a**) Differences between the acoustic impedance (${z}_\text{Si}$) of Si and the effective acoustic impedance (${z}_{eff}$) of the photonic crystals when SiO_2_ filling fraction (${f}_{1s}$) within the unit cell of the photonic crystals is swept from 0 to 0.8 with increments of 0.2 while the value of ${N}_{1}$ is fixed at 8. (**b**) Displacement fields of the two-scale phoxonic cavity for different ${f}_{1s}$. The orange and blue regions represent the SiO_2_ and Si layers, respectively.

The effective acoustic properties of the photonic crystals, whose unit cells have a much smaller length scale than the acoustic wavelength, are obtained by using quasi-static homogenization^32^. The effective mass density is the weighted arithmetic mean of the mass densities of silicon (Si) and silica (SiO_2_), and the effective Young’s modulus is the weighted harmonic mean of the Young’s moduli of Si and SiO_2_, where the weight is the SiO_2_ filling fractions $({f}_{1s})$ of the photonic crystals. Then, we calculate the effective acoustic impedance (${z}_{eff}$), which is the square root of the product of the effective mass density and effective Young’s modulus. Since the effective properties are only influenced by ${f}_{1s}$, we investigate the effective acoustic impedance by sweeping ${f}_{1s}$ from 0 to 0.8 with increments of 0.2. Figure 3a shows the difference between the acoustic impedance (${z}_\text{Si}$) of Si and ${z}_{eff}$ according to ${f}_{1s}$. The difference $\left(\frac{{z}_\text{Si}-{z}_{eff}}{{z}_{2}}\right)$ approaches zero as ${f}_{1s}$ decreases, indicating that the material properties of the photonic crystals become similar to those of Si. We calculate the displacement field according to ${f}_{1s}$ as illustrated in Fig. 3b. The results show that the amplitude of displacement in the cavity increases as the effective property of the photonic crystals becomes similar to the Si.

The modulation of the geometrical constitution of the photonic crystals not only influences the ability to confine acoustic waves but also affects the optical cavity mode frequency. Thus, it is necessary to minimize ${f}_{1s}$ while maintaining the optical cavity mode frequency at 193.5 THz. We found all pairs of ${f}_{1s}$ and ${N}_{1}$ where the photonic crystals confine 1550 nm light by sweeping the geometrical parameters (${N}_{1}$, ${f}_{1s}$) in the range of 1 $\le {N}_{1}\le$ 24 and 0 $\le {f}_{1s}\le$ 1, with increments of 1 and 0.001, respectively. Table 2 shows nine pairs of ${f}_{1s}$ and ${N}_{1}$ to confine 1550 nm light within the phoxonic cavity. When ${N}_{1}$ exceeds 18, size of unit cell (${a}_{1}$) of the photonic crystal becomes much smaller than the wavelength (1550 nm) of light, so the optical cavity mode does not occur at 193.5 THz, the optical frequency corresponding to 1550 nm light.

**Table 2:** The geometric parameters (${N}_{1}$ and ${f}_{1s}$) of the photonic crystals used to confine 1550 nm light in the cavity.

	A1	A2	A3	A4	A5	A6	A7	A8	A9
${N}_{1}$	3	4	5	7	8	9	16	17	18
${f}_{1s}$	0.283	0.234	0.320	0.425	0.306	0.141	0.427	0.343	0.212

**Figure 4:**
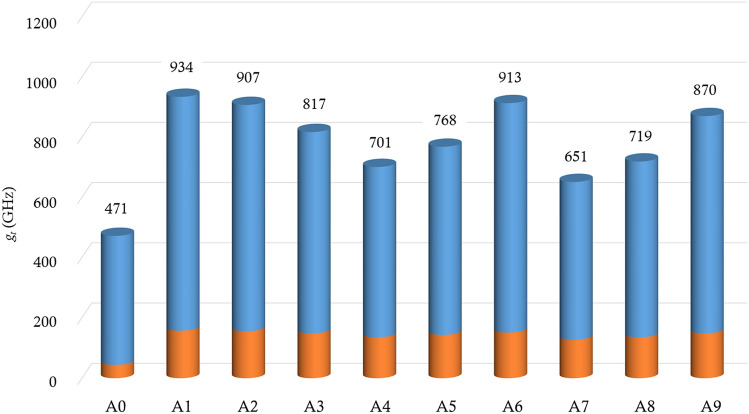
Total frequency modulation (${g}_{t}$) of optical cavity mode for the single-scale phoxonic cavity (A0) depicted in Fig. 1a and the two-scale phoxonic cavities (A1–A9) whose geometrical constitution is depicted in Fig. 1b and their geometrical parameters are listed in Table 2. The blue and orange bars indicate the values of ${g}_{MI}$ and ${g}_{PE}$, respectively.

Figure 4 shows the total modulation (${g}_{t}$) for all cases in Table 2. In the cases A4–A9 where the unit cell size (${a}_{1}$) of the photonic crystal is smaller than half the wavelength, an increase in ${g}_{t}$ is observed as ${f}_{1s}$ decreases. This increase is attributed to the increase in the amplitude of displacement within the cavity. When ${a}_{1}$ is larger than half of the wavelength, ${g}_{t}$ increases as the number of unit cells (${N}_{1}$) decreases. This is because ${a}_{1}$ becomes similar to the wavelength of the infrared light as ${N}_{1}$ decreases, making it suitable for controlling the infrared light. The phoxonic cavity of case A1 achieves the maximum value of ${g}_{t}$ about twice as large as that for the single-scale phoxonic cavity (Case A0).

### Maximization of AO interactions in two-scale phoxonic cavities

In the previous section, we changed the geometrical parameters (${f}_{1s}$, ${N}_{1}$) to enhance the ability to confine acoustic waves in the cavity. To maximize the ability, the effective material properties of the photonic crystals are needed to be the same as those of silicon (Si). However, it is not possible to design such photonic crystals by only changing geometrical parameters. In this section, we adjust the material properties of the photonic crystals using quasi-static homogenization theory^32^ and maximize the AO interactions in the two-scale phoxonic cavities.

According to quasi-static homogenization theory, the effective material properties of the photonic crystals are the same as those of Si when the mass density, Young’s modulus, and acoustic impedance of the Si layers in the photonic crystals are changed to $\rho ^{\prime}$, $E^{\prime}$, and $Z^{\prime}$ as follows:

4 $$\rho ^{\prime}=\frac{{\rho }_\text{Si}-{\rho }_{\text{SiO}_{2}}{f}_{1s}}{1-{f}_{1s}},$$

5 $$E^{\prime}=\frac{1-{f}_{1s}}{\frac{1}{{E}_\text{Si}}-\frac{{f}_{1s}}{{E}_{\text{SiO}_{2}}}},$$

6 $$Z^{\prime}=\sqrt{\rho ^{\prime}E^{\prime}},$$

where ${\rho }_{\text{SiO}_{2}}$ and ${E}_{\text{SiO}_{2}}$ are the mass density and Young’s modulus of silica (SiO_2_) and ${\rho }_\text{Si}$ and ${E}_\text{Si}$ are the mass density and Young’s modulus of Si.

**Figure 5:**
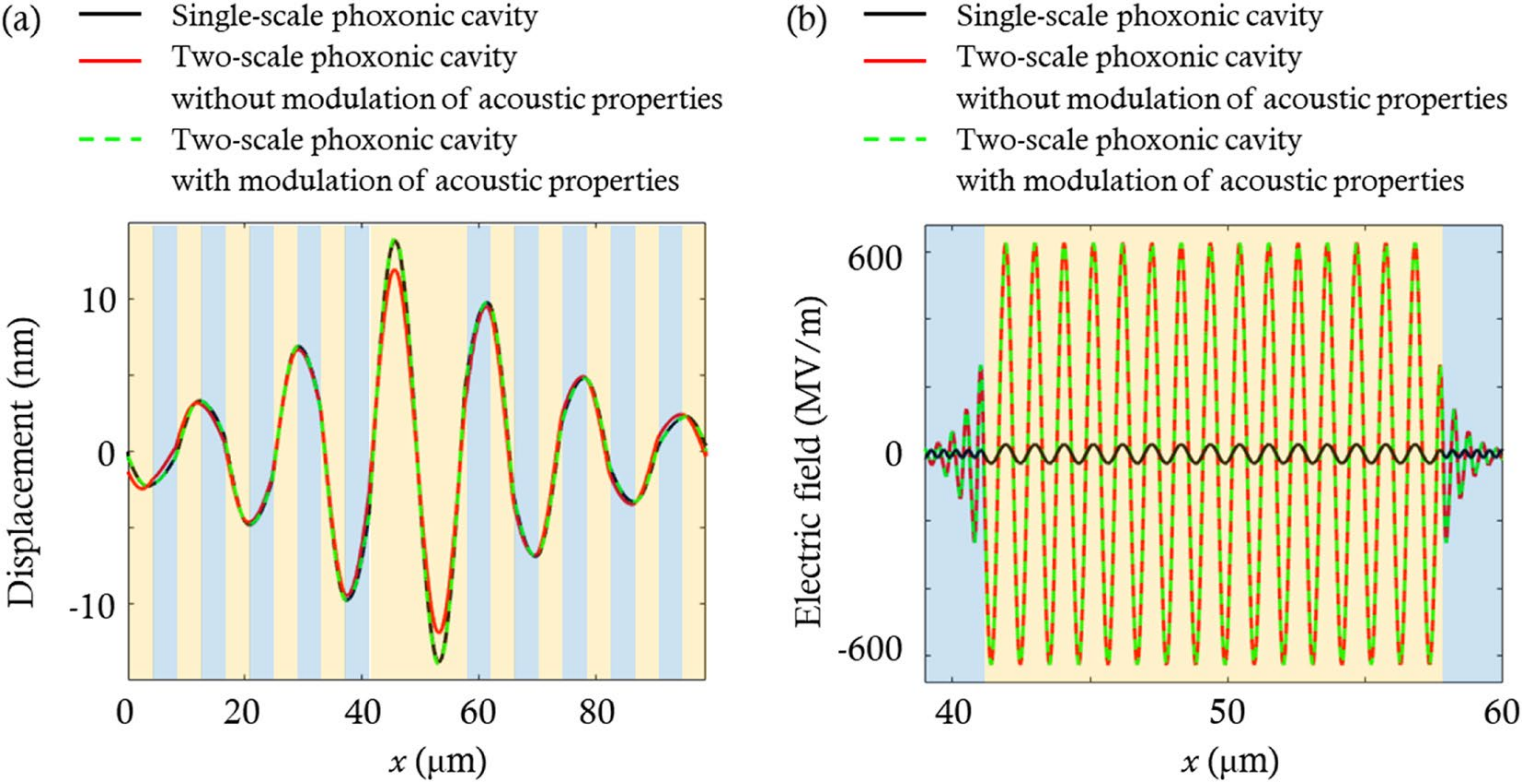
(**a**) Displacement fields at the frequency of the acoustic cavity mode for the phoxonic cavities (solid black and red lines) shown in Fig. 1 and the two-scale phoxonic cavity with change in the material properties of photonic crystals (Green dotted line). (**b**) Electric fields in the three phoxonic cavities at the optical cavity mode frequency. The orange and blue regions represent the SiO_2_ and Si layers, respectively.

Figure 5 shows the displacement and electric fields of the phoxonic cavities (solid black and red lines) depicted in Fig. 1 and the two-scale phoxonic cavity (dotted green line) whose material properties of the Si layers within the photonic crystals are adjusted according to Eqs. (4)–(6). After the adjustment, the broken periodicity of the unit cell of acoustic mirrors is recovered, so the two-scale phoxonic cavity (dotted green line) has the same displacement field as the single-scale phoxonic cavity, as shown in Fig. 5a. In addition, the electric field of the two-scale phoxonic cavity does not change after changing the material properties, as shown in Fig. 5b. Thus, the hypersound is confined more in the cavity and interacts with the infrared light, achieving the total modulation (${g}_{t}$) approximately 18% larger than that before the change of material properties.

**Figure 6:**
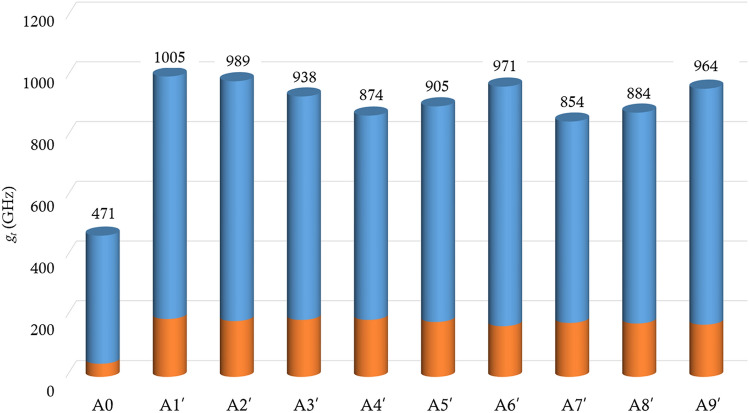
Total frequency modulation (${g}_{t}$) of optical cavity mode for the single-scale phoxonic cavity (A0) depicted in Fig. 1a and the two-scale phoxonic cavities (A1′–A9′) whose geometrical constitution is depicted in Fig. 1b and their geometrical parameters are listed in Table 3. The acoustic properties of the Si layers for photonic crystals are adjusted according to Eqs. (4)–(6). The blue and orange bars indicate the values of ${g}_{MI}$ and ${g}_{PE}$, respectively.

We further enhance the AO interaction by not only adjusting material properties of photonic crystals but also tuning their geometrical parameters. We change the geometrical parameters (${N}_{1}$ and ${f}_{1s}$) of the photonic crystals whose effective material properties are adjusted to be the same as those of Si. ${N}_{1}$ and ${f}_{1s}$ are swept in the range of 1 $\le {N}_{1}\le$ 24 and 0 $\le {f}_{1s}\le$ 1, with increments of 1 and 0.001, respectively. Table 3 shows all combinations of geometrical parameters of the photonic crystals and the acoustic impedance of Si layer (${N}_{1}$, ${f}_{1s}$, $Z^{\prime}$) for confining 1550 nm light in the cavity. Since the optical properties of the photonic crystals remain the same, the pairs of ${f}_{1s}$ and ${N}_{1}$ in Table 3 are the same as those in Table 2. For case A1′–A9′, we obtain the total modulation (${g}_{t}$), as shown in Fig. 6, and the value of ${g}_{t}$ are approximately 12–43% larger than those in Fig. 4. Among the cases (A1′–A9′), A1′ exhibits the maximum value of ${g}_{t}$, and this modulation value is 2.1 times that of a single-scale phoxonic cavity (A0).

**Table 3:** The geometrical parameters (${N}_{1}$, ${f}_{1s}$) and the acoustic impedance ($Z^{\prime}$) photonic crystals to confine 1550 nm light in the cavity.

	A1′	A2′	A3′	A4′	A5′	A6′	A7′	A8′	A9′
${N}_{1}$	3	4	5	7	8	9	16	17	18
${f}_{1s}$	0.283	0.234	0.320	0.425	0.306	0.141	0.427	0.343	0.212
$Z^{\prime}/{10}^{6}$ $(Rayls)$	26.5	24.4	28.8	47.5	27.8	21.8	48.4	30.8	23.6

## Conclusions

In this study, we proposed a two-scale phoxonic cavity by employing the concept of structural hierarchy to enhance AO interaction. For single-scale phoxonic cavities, existing studies^16–18^ have examined interactions between infrared light and high-frequency (a few GHz) sound with the same wavelength scale. Since the high-frequency sound was highly attenuated in dissipative media, weakening AO interaction, one must use sound at low frequencies (a few hundred of MHz) whose attenuation coefficient is negligible. Unlike a single-scale phoxonic cavity, the two-scale phoxonic cavity could confine both low-frequency sound and infrared light with different wavelengths. To quantitatively examine AO interactions in the phoxonic cavities, we calculated the total modulation (${g}_{t}$) of the optical cavity mode frequency. The results showed that ${g}_{t}$ in the two-scale phoxonic cavity was approximately 1.6 times than that in the single-scale phoxonic cavity. To further enhance the AO interaction in the two-scale phoxonic cavity, we modified the geometrical constitution and material properties of the photonic crystals using quasi-static homogenization theory to tune effective material properties of the photonic crystals more similar to those of Si. This modified phoxonic cavity exhibited ${g}_{t}$ 2.1 times larger than that in the conventional phoxonic cavity. Thus, the present study shows that the proposed phoxonic cavity with structural hierarchy can be a good candidate for enhancement of AO interaction.

## Data Availability

All data generated or analyzed during this study are included in the manuscript.
